# Factors That Influence Base-Catalyzed Thiol-Ene Hydrogel Synthesis

**DOI:** 10.3390/gels9110917

**Published:** 2023-11-20

**Authors:** Nolan Morrison, Brandon M. Vogel

**Affiliations:** Department of Chemical Engineering, Bucknell University, Lewisburg, PA 17837, USA; njm015@bucknell.edu

**Keywords:** hydrogels, injectable, erodible, thiol-ene, ETTMP, PEGDA

## Abstract

Injectable, localized drug delivery using hydrogels made from ethoxylated trimethylolpropane tri-3-mercaptopropionate (ETTMP) and poly(ethylene glycol) diacrylate (PEGDA) has shown great potential due to these hydrogels’ ability to exhibit non-swelling behavior and tunable drug release properties. However, current synthesis methods in the literature suffer from poor ETTMP solubility in water, slow gelation times exceeding 20 min, and a lack of reproducibility. To address these limitations, we have developed a reliable synthesis procedure and conducted a sensitivity analysis of key variables. This has enabled us to synthesize ETTMP-PEGDA hydrogels in a polymer concentration range of 15 to 90 wt% with gelation times of less than 2 min and moduli ranging from 3.5 to 190 kPa. We overcame two synthesis limitations by identifying the impact of residual mercaptopropionic acid and alumina purification column height on gelation time and by premixing ETTMP and PEGDA to overcome low ETTMP solubility in water. Our ETTMP-PEGDA mixture can be stored at −20 °C for up to 2 months without crosslinking, allowing easy storage and shipment. These and previous results demonstrate the potential of ETTMP-PEGDA hydrogels as promising candidates for injectable, localized drug delivery with tunable drug release properties.

## 1. Introduction

### 1.1. Injectable Hydrogels for Drug Delivery

Hydrogels are three-dimensional networks of crosslinked polymers swollen with water [1]. Their composition can be tuned to mimic the hydrophilic nature of human tissues, making them particularly suitable for biomedical applications [2,3,4,5]. The biocompatibility of many hydrogels has positioned them as potentially useful materials for drug delivery, along with their degradation properties that can be finely tuned and their mechanical characteristics, which may closely resemble those of human tissues [6,7,8,9,10,11,12,13,14,15,16,17,18,19]. The ability to precisely target specific body areas and provide controlled release of therapeutic agents via these hydrogels significantly minimizes the potential for toxic or undesired systemic side effects, a common concern in more traditional drug delivery methods [20,21,22].

There are several ways to achieve crosslinking, including external sources such as electron beam-induced crosslinking [23] or ultraviolet (UV) photopolymerization, a process that generates free radicals to initiate the crosslinking of polymers [24,25,26,27,28,29,30]. The UV method, while effective, faces limitations, particularly in in vivo applications. The requirement for external UV light sources to initiate the crosslinking polymerization process makes it less viable for direct internal use without prior implantation, thus limiting its practicality in certain clinical scenarios.

Alternative crosslinking methods have been explored to overcome these challenges, with chemically induced crosslinking as a promising technique. This method allows for directly introducing a drug-loaded hydrogel into the body, where it can gradually release its payload over an extended period. A notable advancement in this area is thiol-ene crosslinking, a reaction that can rapidly gel in the body [21,24,31,32,33,34,35,36,37]. This process occurs without external stimuli such as light, making it a highly efficient and practical approach for in vivo applications. Developing these injectable hydrogels, which are capable of in situ formation and controlled drug release, opens new avenues in patient-specific therapy, potentially improving the efficacy of treatment regimens.

### 1.2. ETTMP-PEGDA Hydrogels

**Figure 1:**
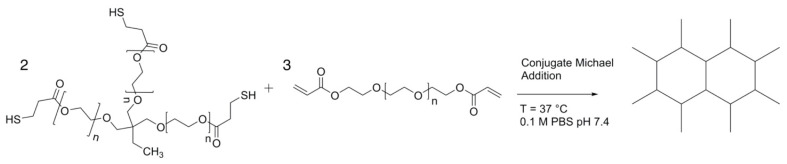
Conjugate Michael reaction to form the thiol–acrylate crosslinking network. A 2:3 ratio of ETTMP to PEGDA is used to achieve stoichiometric equivalence.

This paper focuses on the thiol-ene hydrogel system composed of trifunctional thiol, ethoxylated trimethylolpropane tri-3-mercaptopropionate (ETTMP), and alkene poly(ethylene glycol) diacrylate (PEGDA). This system can form hydrogels with a base-catalyzed conjugate Michael addition reaction, where thiol–acrylate reactions form the crosslinked network (Figure 1). ETTMP-PEGDA hydrogels also exhibit non-swelling behavior at equilibrium compositions, enabling injections into pressure-sensitive tissues or joints [38]. Michael addition reaction kinetics are dependent on reaction conditions, allowing for tunable hydrogel properties to match the desired application [35,39].

Recently, while working on a paper on these hydrogels, we discovered that the literature methods to make these hydrogels were incomplete [32]. Several research groups have used Michael addition thiol-ene polymerization ETTMP-PEGDA hydrogels, each with different synthesis protocols and reaction conditions. We briefly summarize these conditions below to provide context regarding the current protocols and the variability in reaction times.

Pritchard et al. first dissolved PEGDA (M_w_ = 400 g/mol) and ETTMP in individual phosphate-buffered saline (PBS) containers for 24 h at 4 °C. Following initial precursor preparation, the appropriate amount of each solution was added to a Falcon tube to achieve stoichiometric equivalence. After a 5 min incubation at 4 °C, the solution was then maintained at 37 °C, resulting in a gelation time of about 5 min. The hydrogel polymer concentration was held at 25 wt%, and the ETTMP stock solutions could not exceed 45 wt% because of poor water solubility at 37 °C [36].

Khan et al. developed a protocol where PEGDA (M_w_ = 700 g/mol) and ETTMP were added to the extracellular buffer (ECB) along with 2 M NaOH to initiate the reaction, as opposed to PBS. Additionally, hydrogels were made with much lower polymer concentrations (8.5, 9, and 9.5 wt%) and, in some cases, with off-stoichiometric thiol-to-acrylate molar ratios (1 and 1.05). Synthesis occurred at room temperature with resulting gelation times between 25 and 150 min [40].

Moon et al. used a range of PEGDA (M_w_ = 260, 510, and 670 g/mol) with ETTMP for their formulation. First, ETTMP was added to a 0.1 M solution of aqueous NaHCO_3_ and stirred. After adding PEGDA and mixing, the hydrogel solution was allowed to sit for 1 h to complete gelation. Neither Khan et al. nor Moon et al. included a precursor purification step before synthesis [41].

In our attempts to reproduce these results, the above methods resulted in irreproducible, time-inefficient synthesis or, in the worst cases, failed to produce a hydrogel. Here, we describe a robust and reproducible ETTMP-PEGDA hydrogel synthesis protocol. This protocol can be used to prepare hydrogels in the range of 15 wt% to over 90 wt% polymer concentration. We determine the effect of precursor purification, buffer pH, mixing type, and storage conditions on the hydrogel gelation time and mechanical properties. Our results provide critical guidance for obtaining the desired gelation time or mechanical properties to meet the needs of injectable hydrogel applications. Additionally, these methods are expected to apply to other base-catalyzed Michael addition hydrogel systems.

## 2. Results and Discussion

### 2.1. Premixing ETTMP-PEGDA Improves Aqueous Solubility and Enables Hydrogel Compositions over a Large Range

Previous methods reported in the literature for synthesizing ETTMP-PEGDA hydrogels involved dissolving the components in separate buffer solutions and then mixing them together before placing the mixture at 4 °C for 24 h to ensure intimate mixing due to the improved solubility of ETTMP at lower temperatures [20,21]. However, these methods were limited by the poor solubility of ETTMP in water and buffer solutions at 37 °C, which restricted the range of hydrogel compositions that could be made. We developed a new method where ETTMP and PEGDA are premixed before adding the buffer, which overcomes the solubility limitation of ETTMP and allows hydrogels to be made at a wider range of polymer concentrations up to 90 wt%. Our method significantly improves reproducibility and enables a wide range of gelation times with easy tunability. Gelation can be achieved at physiologically relevant pHs without the need for an additional 24 h incubation at 4 °C after mixing. Table 1 shows the effect of polymer concentration on hydrogel mechanical properties, with increasing polymer concentration resulting in nearly two orders of magnitude change in the equilibrium modulus and crosslink density. Frequency sweep rheological tests were run on the 25 wt% and the 35 wt% samples. The hydrogel with 35 wt% concentration generally exhibits higher moduli (both G′ and G″) and viscosity, which is consistent with the expectation that increasing the polymer concentration in a hydrogel increases these mechanical properties due to the higher degree of crosslinking. The magnitude of the storage modulus G′ at lower frequencies is higher for the 35 wt% hydrogel, indicating a stronger or more crosslinked network than the 25 wt% hydrogel. The complex viscosity is higher across the frequency range for the 35 wt% hydrogel, possibly due to the higher concentration of polymer chains that contribute to an increased resistance to flow. The frequency sweep data and all of the time sweep data figures are provided in the Supplementary Data Figure S1a–S1g.

### 2.2. Sensitivity Analysis of the Experimental Conditions’ Effects on the Rheological Properties of Hydrogels

We conducted dynamic rheology to quantify the effect of purification column height, buffer pH, and mixing type on the hydrogel gelation time and mechanical properties. We found that the crosslink density, mesh size, and equilibrium modulus were not a strong function of PCH, buffer pH, or the type of mixing once the reaction reached equilibrium. Rheological data of these mechanical properties can be found in the Supporting Information. Only the hydrogel polymer concentration was found to impact the mechanical properties (Table 1). The result agrees with previous literature pointing to a high conversion of thiols and acrylates typical of thiol-ene polymerizations.

#### 2.2.1. Purification Column Height Controls the Final Gelation Time

**Figure 2:**
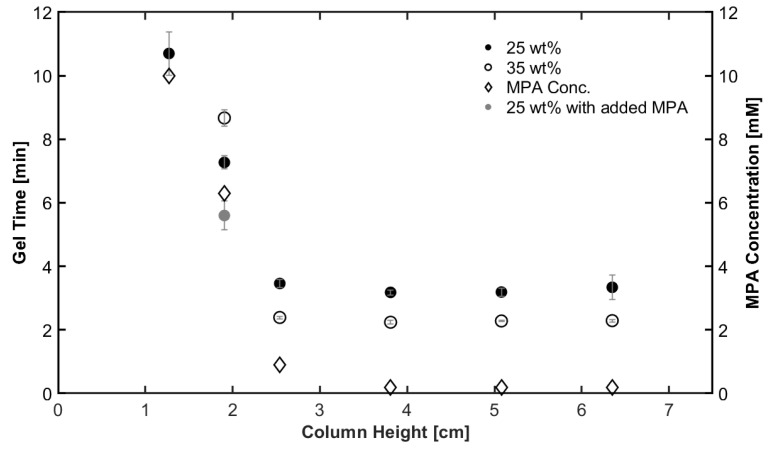
Increasing the purification column height reduces the gelation time and MPA concentration. All samples were made using a buffer pH of 7.4 and a 15 s vortex to mix. Error bars represent standard deviation, where n = 3.

Purification column height substantially affected the hydrogel gelation time for both 25 wt% and 35 wt% hydrogel concentrations. Gelation time remained relatively constant for purification column heights in the range of 2.54 to 6.35 cm (Figure 2). In this range, the 25 and 35 wt% hydrogels demonstrated gelation times of around 3.3 and 2.3 min, respectively. When the PCH was lowered to 1.27 cm, the gelation time drastically increased to 10.7 min for 25 wt% and 13 min for 35 wt%. Insufficient purification leaves higher concentrations of monofunctional MPA, resulting in the consumption of acrylates that do not contribute to the growing network structure and a change in local pH, yielding a slower reaction rate. This result demonstrates that the removal of inhibitors is essential in optimizing hydrogel synthesis. The absence of ETTMP purification resulted in samples that did not cure in our tests at 0.1 M pH 7.4 using PBS buffer.

Once the PCH was increased above 2.54 cm, the residual concentration of MPA decreased, which correlates with increasing observed with gel time. At a PCH of 3.81 cm and above, the MPA concentration was 0.18 mM. We quantified that at PCH values of 0 and 0.635 cm, the MPA concentrations were about 81 and 38 mM, respectively. No gelation occurred at a PCH of 0 or 0.635 cm, where the residual MPA concentration is too great and completely inhibits the thiol Michael reaction.

Although the MPA concentration in ETTMP provides insight into the relationship between PCH and gel time, we wanted to determine how the gel time was affected. To this end, ETTMP was added to PBS and PBS-containing PEGDA, and the pH was measured to identify a change in pH (Table 2). We found that adding impure ETTMP led to a much more acidic solution (pH 4.06 before adding PEGDA and 4.25 after adding PEGDA). Purified ETTMP (3.81 cm column height) had a pH of 7.08 before adding PEGDA and 7.4 after adding PEGDA, a significant shift of 3.15 pH units to the physiological pH range. The significant change in local pH caused an order of magnitude increase in the base-catalyzed thiol Michael gelation time. Finally, we note that increasing the column height beyond 3.81 cm did not result in a significant change in pH or gelation time.

We note that in several previous publications, the synthesis methods did not specify any purification details of ETTMP, or the authors used high-solution pH to achieve curing [40,41]. Thus, one method to overcome the high MPA concentration would be to add an excess of the base to the reaction. Indeed, additional base added to the formulation may be a method to reduce the purification time needed to remove MPA.

**Figure 3:**
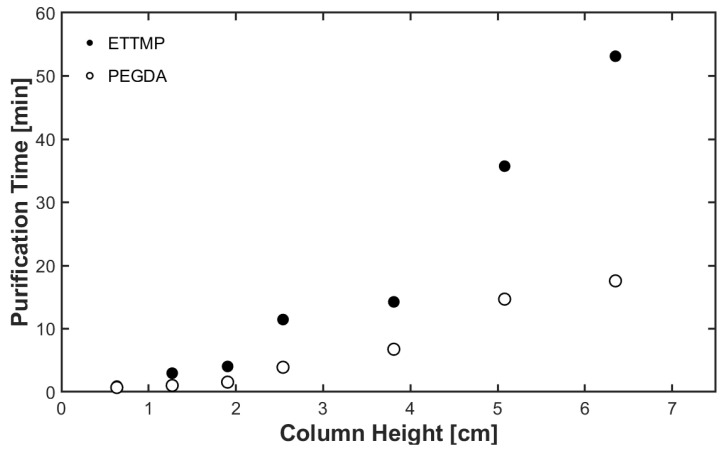
Time required to purify ETTMP and PEGDA at various purification column heights (PCHs).

Not only does the PCH affect the gelation time and MPA concentration, but it also affects the time required to purify the ETTMP and PEGDA (Figure 3). Purification time for ETTMP ranged from 3 to 52.1 min, and was 1 to 17.6 min for PEGDA. We found the optimal ETTMP PCH to be 3.81 cm of basic alumina. A break-through curve was attempted at an alumina column height of 3.81 cm, but after 8x the mass of alumina in ETTMP was passed through the column, the flow rate slowed considerably with little effect on the concentration of MPA in the effluent.

#### 2.2.2. Buffer pH Is Another Parameter to Control Gelation Time

**Figure 4:**
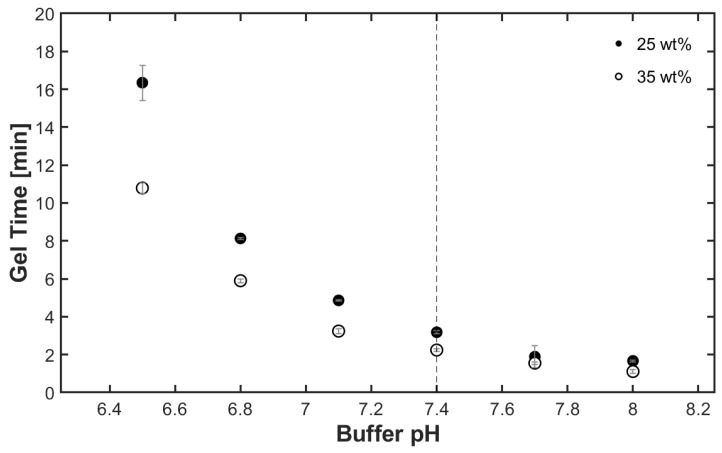
Higher pH and a 35 wt% gel formulation resulted in faster gelation times. Error bars represent standard deviation, where n = 3.

A series of 25 and 35 wt% polymer concentration hydrogels were prepared with PBS adjusted to pH values between 6.5 and 8 to determine the effect of buffer pH on gelation time. Figure 4 displays the gelation times determined via dynamic rheology as a function of buffer pH. Both polymer concentrations demonstrated a non-linear increase in gelation time as buffer pH was decreased. This result agrees with the outcome of a similar study conducted by Pritchard et al., where the gelation time decreased exponentially with increased buffer pH [36]. At higher pH, the difference in gelation time attributed to polymer concentration was minimal (0.56 min difference at pH 8), but the gelation time difference between the two hydrogel polymer concentrations became exaggerated as pH decreased (5.56 min difference at a pH of 6.5). The difference in gelation time is due to an increased concentration of reactive species in the presence of the base, allowing for more rapid crosslink formation [35]. The correlation between pH, polymer concentration, and gelation time allows easy hydrogel tunability for various applications. However, for most in vivo applications, a pH of 7.4 is the physiologically relevant condition, and this pH yielded rapid gelation times (2 to 3 min).

#### 2.2.3. Vortex Mixing Results in Faster Gelation Times When Compared to Hand Mixing

Sufficient mixing of the precursor-buffer solution is critical to reproducibly synthesize ETTMP-PEGDA hydrogels. We tested mixing times between 5 and 25 s and mixing types, namely hand mixing versus vortex mixing, and quantified their impact on gelation time with dynamic rheology. Table 3 highlights the results for both 25 and 35 wt% hydrogels. For both hydrogel samples, vortexing yielded faster gelation times compared to shaking by hand. Furthermore, increasing the vortex time by 10 s decreased the gelation time by 0.13 min for 25 wt% and 0.26 min for 35 wt%, on average. When we increased the hand mixing time by 5 s, the gelation increased reproducibly. We assumed that a longer degree of mixing would result in faster gelation up to a point due to the thorough mixing of the ETTMP, PEGDA, and buffer solution. We attribute the increased gelation time with increased hand mixing to the inherent inconsistency of hand mixing; maintaining a constant shaking rate and force is nearly impossible. For this reason, and slightly faster gelation times, vortexing for 15 s is deemed practical for this hydrogel system. Increasing the vortex time over 15 s showed minimal improvements and introduced more inconsistency in the gelation time data.

### 2.3. ETTMP-PEGDA Can Be Stored Frozen for Longer Than Two Months without Changes to Gelation Time

**Figure 5:**
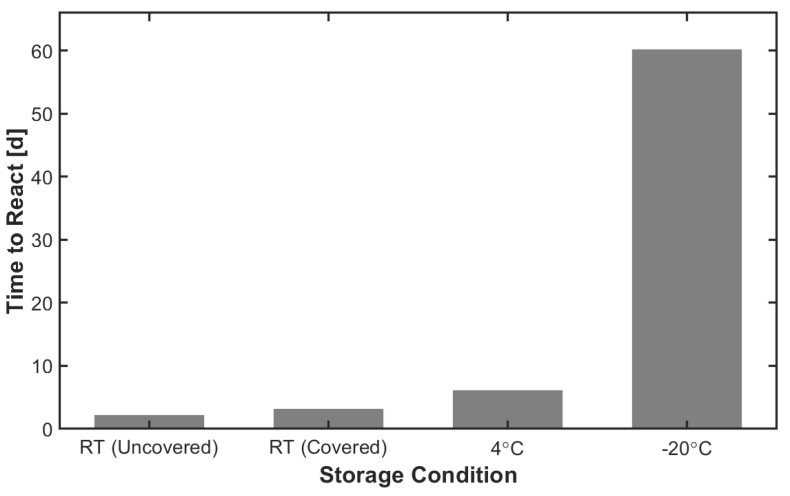
Storing stoichiometric mixtures of ETTMP and PEGDA at −20 °C prevents unwanted storage crosslinking for at least 60 d compared to storing in a refrigerator and at 25 °C.

**Figure 6:**
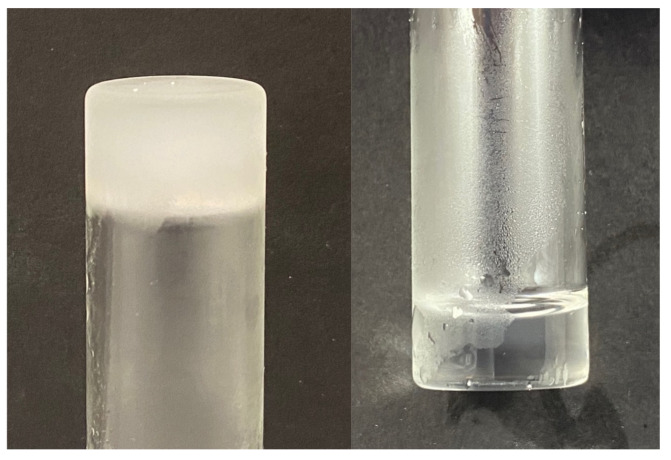
A stoichiometric mixture of ETTMP and PEGDA froze after being stored at −20 °C (**left**) and thawed at room temperature (**right**).

Easily stored and reconstituted mixtures of the precursors are important for practical clinical use. When stored separately, the precursors remain stable at the conditions recommended by the manufacturer (ETTMP at room temperature and PEGDA at 4 °C). However, identifying conditions where premixed ETTMP and PEGDA formulations could be stored for a prolonged period is important for clinical applications. To this end, stoichiometric amounts of ETTMP and PEGDA were added and mixed in a vial under yellow light (UV filtered) to reduce the formation of free radicals. Samples were then stored at room temperature exposed to ambient light or in the absence of light at room temperature, 4 °C, or −20 °C. Samples were visually inspected daily to determine if the ETTMP-PEGDA mixture had reacted. This was performed by tilting the vial to determine if the contents flowed or were gelled. Figure 5 shows the number of days samples of different experimental storage conditions took to gel (when the vial contents stopped flowing and formed a gel-like material). Storage temperature was shown to have a greater impact on preventing reaction than protection from UV light. That is, slowing the reaction kinetics had a greater effect than limiting free radical generation. Precursor mixtures stored in the absence of light at room temperature only lasted 1 d longer than those exposed to light (3 d versus 2 d). Reducing the temperature to 4 °C extended the storage period to 6 d. Samples stored at −20 °C froze solid (Figure 6) and remained unreacted for about 2 months.

We hypothesize that the freezing of the ETTMP-PEGDA mixture significantly reduces the reaction kinetics, increasing the time for gelation. Frozen samples thaw after roughly 5 min at room temperature and can then be used to synthesize a hydrogel with the addition of 0.1 M PBS. We used DSC to determine the freezing points, if any, of the ETTMP, PEGDA, and mixture. The results show that PEGDA displays two freezing point peaks at −15.9 °C and −20.7 °C (Figure S9). This is due to PEGDA being a semicrystalline polymer that can form crystals of various sizes and structures during the liquid–solid phase transition—a phenomenon Cheng et al. investigated using low-molecular weight poly(ethylene oxide) (PEO) with various end groups, resulting in similar semicrystalline polymers [42]. Within the range of 35 °C to −50 °C, ETTMP did not display a freezing point. Upon mixing the two components, a peak at −23.6 °C was found, and the freezing point was determined to be in the range of −17.9 °C to −27.2 °C. A shoulder peak also occurred at −8.5 °C. Comparing the PEGDA and mixture results shows a freezing point depression of about 7.7 °C for both peaks with ETTMP as an impurity.

## 3. Conclusions

This paper provides a robust procedure to synthesize ETTMP-PEGDA hydrogels reproducibly and discusses the factors that influence successful synthesis. Hydrogels can be formulated within a wide range of polymer concentrations, allowing for tunability of the gelation time and mechanical properties. The purification column height directly affects the amount of MPA impurity in the ETTMP and has a substantial effect on gelation time. Furthermore, the thiol Michael addition reaction kinetics displayed a non-linear relationship with the PBS buffer pH used in the formulation. Finally, long-term storage of ETTMP-PEGDA in the absence of a solvent is possible when kept at −20 °C, further expanding the scope of potential clinically relevant applications for the hydrogels.

## 4. Materials and Methods

### 4.1. Materials

Poly(ethylene glycol) diacrylate (Mn = 575 g/mol) (PEG-575-DA, Opalescence, Warrington, PA, USA) was purchased from Sigma Aldrich, St. Louis, MO, USA. Ethoxylated trimethylolpropane tri-3-mercaptopropionate (THIOCURE, ETTMP 1300) was donated by Bruno Bock (Marschacht, Germany). Brockmann I basic aluminum oxide (150 µm particle diameter) was purchased from Sigma Aldrich, St. Louis, MI, USA. To prepare the 0.1 M phosphate buffered saline (PBS) solution, 8 g sodium chloride (JT Baker, Phillipsburg, NJ, USA), 0.2 g potassium chloride (VWR Chemicals BDH, Radnor, PA, USA), 1.44 g sodium phosphate dibasic (VWR Chemicals BDH, Radnor, PA, USA), and 0.24 g potassium phosphate monobasic (VWR Chemicals BDH, Radnor, PA, USA) were dissolved in deionized (DI) H_2_O and diluted to 1 L. The pH was then adjusted using NaOH or HCl after dilution.

### 4.2. Instrumentation

The thermal properties and freezing point of a mixture of ETTMP and PEGDA were assessed using DSC to gain insight into storage conditions. For DSC measurements, stoichiometrically equivalent amounts of each precursor were added to a vial and vortexed for 15 s without PBS. Roughly 3.5 to 6.5 mg of the mixture was pipetted and sealed in TZero aluminum pans with a hermetic lid (TA Instruments, New Castle, DE, USA) before being placed into the sample tray of a Q2000 DSC (TA Instruments, New Castle, DE, USA). Samples were initially equilibrated at 35 °C and then cooled to –50 °C at a rate of 10 °C/min. The temperature corresponding with the maximum value of the heat flux peak was determined to be the freezing point of the sample. Individual samples of ETTMP and PEGDA were measured as controls for each component.

Hydrogel rheological measurements were performed with a Discovery Hybrid Rheometer HR-2 (TA^®^ Instruments, New Castle, DE, USA) with a Peltier plate (TA^®^ Instruments), allowing the plate’s temperature to be monitored and adjusted. A 0° parallel plate (TA^®^ Instruments) was used for all experiments. The Peltier plate was set to the desired temperature and allowed to equilibrate before zeroing the gap. We used dynamic rheology to determine gelation times, measure equilibrium modulus, and estimate crosslink density and mesh size within the context of a model described below. The gelation time was defined as the intersection of the storage modulus (G′) and the loss modulus (G″). The equilibrium modulus refers to the value at which the storage modulus plateaus. Crosslink density (ν) was calculated using Equations (1) and (2) [43]:

(1) $$G^{*}=\nu RT$$

where R is the gas constant, T is temperature, and G^*^, the complex modulus, is:

(2) $$G^{*}=\sqrt{{G\prime }^{2}+{G\prime \prime }^{2}}$$

Mesh size (r_mesh_) was calculated via the classical theory of rubber elasticity, which relates mesh size to shear modulus (G) by [44]:

(3) $$r_{mesh}={\left(\frac{6RT}{\pi N_{Av}G}\right)}^{1/3}$$

where N_Av_ is Avogadro’s number.

Hydrogel solutions were prepared as described in Section 4.4, and 490 μL was transferred directly to the rheometer. There were roughly 35 s between vortexing and starting the data collection. A time sweep was conducted as per the parameters in Table 4 to collect the raw data required to compute the hydrogel properties mentioned above. For an example, storage and loss moduli vs. time plot, refer to the Supporting Information. A frequency sweep was conducted to ensure that the time sweep conditions were within the linear viscoelastic region of the hydrogel, the region of testing where the sample structure was not destroyed.

Three separate studies were conducted using dynamic rheology to isolate major variables in the hydrogel synthesis protocol and determine their impact on successful gelation and mechanical properties. Purification column height (PCH), buffer pH, and mixing type were identified as the variables of interest. In the first study, the height of aluminum oxide in the inhibitor removal column was tested at values of 0, 0.635, 1.27, 1.905, 2.54, 3.81, 5.08, and 6.35 cm. Next, PBS solutions with pH values of 6.5, 6.8, 7.1, 7.4, 7.7, and 8 were prepared and tested. Finally, in the third study, the following mixing methods were tested: 5 s by hand, 10 s by hand, 15 s vortex, and 25 s vortex. Unless specified for the given study, the PCH, buffer pH, and mixing type were constant at 3.81 cm, 7.4, and 15 s vortex, respectively. Hydrogels with 25 and 35 wt% polymer concentrations were used for each study.

### 4.3. ETTMP and PEGDA Hydrogel Precursor Purification

**Figure 7:**
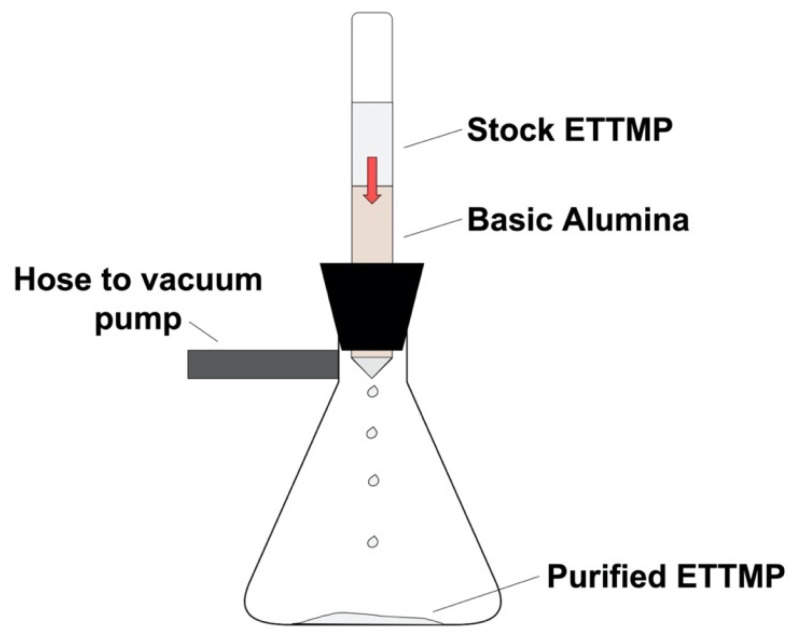
Purification schematic using basic alumina as the adsorbent. Note that the red arrow represents the flow of ETTMP through the column of basic alumina to remove degraded MPA.

We separately purified poly(ethylene glycol) diacrylate (PEGDA) and ethoxylated trimethylolpropane tri-3-mercaptopropionate (ETTMP) before use. Basic alumina was added to a 6-inch-long glass column plugged with glass wool up to a specified column height. The thiol or acrylate was added to the top of the column and pulled through the column with a vacuum pump (Δp = 4.67 kPa) (Figure 7). The inhibitor hydroquinone monomethyl ether (MEHQ) was removed from PEGDA and degraded mercaptopropionic acid (MPA) was removed from ETTMP. The quantity of ETTMP and PEGDA added to the column was twice the mass of the aluminum oxide. Purified PEGDA and ETTMP were collected in separate scintillation vials and stored at 4 °C to limit the rate of ester degradation. We recommend wrapping the vial containing purified PEGDA in aluminum foil to prevent free radical generation and consumption of the acrylates. We ran a range of aluminum oxide column heights (0 to 6.35 cm) for purification to determine the column height effect on hydrogel synthesis. To purify larger batches, we kept the column height the same but increased the column diameters to maintain the 2:1 alumina to precursor ratio. We tried to determine the breakthrough curves for MPA based on alumina column height, but due to possible curing of ETTMP within the column, the alumina column became fouled, leading to a reduction in the flow of ETTMP and eventually stopping it before MPA breakthrough could be achieved.

### 4.4. Hydrogel Synthesis

ETTMP-PEGDA hydrogels were synthesized with a base-catalyzed conjugate Michael addition in 0.1 M PBS buffer (Figure 1). To a vial containing 0.1 M PBS, ETTMP and PEGDA were added in a 2:3 thiol-to-acrylate molar ratio to maintain stoichiometric equivalence. The amount of PBS added was tuned to yield the desired total polymer weight percentage. For example, to fabricate a 2 g hydrogel with a 25 wt% polymer concentration, 0.260 mL (0.234 mmol) of ETTMP and 0.180 mL (0.351 mmol) of PEGDA were added to 1.5 mL of 0.1 M PBS. The ETTMP-PEGDA-PBS mixture was then vortexed for 15 s. Gelation occurred directly on the parallel plate and took between 2 and 63 min, depending on the hydrogel formulation. This procedure was used for all experiments unless specified otherwise.

### 4.5. Determining Mercaptopropionic Acid Concentration in ETTMP

Mercaptopropionic acid (MPA) is a degradation product of ETTMP that is formed by hydrolysis of the ester bonds in ETTMP. A weak acid–strong base titration method was used to assess the concentration of MPA present in ETTMP following purification at various column heights. A 0.09 M solution of ETTMP in deionized H_2_O was prepared in a beaker, and a 0.1 M NaOH solution was titrated in 0.1 mL increments until the first equivalence point was observed. The pH of the solution was analyzed using a digital pH probe and recorded after each addition of NaOH. The first equivalence point represents the amount of base required to neutralize the MPA completely. With a 1:1 acid–base reaction and a known volume of base added, the concentration of MPA was calculated. This protocol was conducted for samples of ETTMP purified at column heights ranging from 0.635 to 5.08 cm. A full titration was completed for impure ETTMP (PCH = 0 cm) and purified ETTMP (PCH = 6.35 cm), which can be found in the Supporting Information.

### 4.6. Quantifying the Changes in Local pH Due to Mercaptopropionic Acid

We quantified the change in pH caused by the presence of MPA because the presence of additional hydrogen ions affected the gelation kinetics. Briefly, 4.3 mL of ETTMP was added to a beaker containing 25 mL of 0.1 M PBS (pH 7.4). After recording the pH, 3 mL of PEGDA was added to the beaker to determine any effect PEGDA may have on the system pH. The pH was quickly recorded before gelation occurred. This final pH represents the pH of a 25 wt% hydrogel before curing. This protocol was conducted for samples of ETTMP and PEGDA purified at alumina column heights ranging from 0 to 6.35 cm.

## Figures and Tables

**Table 1:** Mechanical properties of hydrogels synthesized with a range of polymer concentrations. All properties were found using dynamic rheology. Error is represented by standard deviation, where n = 3. Conditions: buffer pH of 7.4 and a PCH of 3.81 cm.

Polymer Concentration (wt%)	Gelation Time (min)	Crosslink Density (mol/m^3^)	Mesh Size (nm)	G′ (kPa)	G″ (kPa)	Tan δ
15	4.76 ± 0.78	1.43 ± 1.16	14.40 ± 3.39	3.48 ± 2.85	0.019 ± 0.0034	0.0074 ± 0.0035
25	3.18 ± 0.08	6.94 ± 2.03	7.82 ± 0.78	16.91 ± 4.94	0.0525 ± 0.0024	0.0033 ± 0.0008
35	2.24 ± 0.06	20.35 ± 3.39	5.42 ± 0.31	49.57 ± 8.23	0.1248 ± 0.0066	0.0026± 0.0003
90	63.57 ± 16.75	77.22 ± 23.49	3.51 ± 0.32	188.80± 57.69	9.43 ± 1.11	0.0525 ± 0.0141

**Table 2:** The resulting pH of ETTMP when added to PBS (pH 7.4) and PBS containing PEGDA when purified at various column heights for a 25 wt% hydrogel formulation.

Column Height (cm)	pH of ETTMP in PBS	pH of ETTMP in PBS + PEGDA
0	4.06	4.25
0.635	5.43	5.64
1.27	6.84	7.00
1.905	6.87	7.02
2.54	7.02	7.22
3.81	7.08	7.40
5.08	7.07	7.41
6.35	7.09	7.43

**Table 3:** Gelation times for 25 and 35 wt% hydrogels upon implementing different mixing times and types. Error is represented by standard deviation, where n = 3.

Mixing Time (s)	Mixing Type	25 wt% Gelation Time (min)	35 wt% Gelation Time (min)
5	Hand	3.34 ± 0.03	2.47 ± 0.12
10	Hand	3.74 ± 0.04	2.50 ± 0.06
15	Vortex	3.18 ± 0.08	2.24 ± 0.06
25	Vortex	3.05 ± 0.33	1.98 ± 0.08

**Table 4:** Summary of the rheological parameters for time and frequency sweep experiments using parallel plate geometry with gel samples 25 mm in diameter and 1 mm in height.

Test Type	Frequency (s^−1^)	Strain (%)	Points Per Decade
Time Sweep	10	5	N/A
Frequency Sweep	0.01 to 100	5	5

## Data Availability

The raw data required to reproduce these findings are available to download from [INSERT PERMANENT WEB LINK(s)]. The processed data required to reproduce these findings are available to download from [INSERT PERMANENT WEB LINK(s)].

## Supplementary Materials

The following supporting information can be downloaded at: https://www.mdpi.com/article/10.3390/gels9110917/s1.
