# Supplementary material for: Factors That Influence Base-Catalyzed Thiol-Ene Hydrogel Synthesis
Source: Gels. 2023 Nov 20;9(11):917. doi: 10.3390/gels9110917 (PMC10671550; doi:10.3390/gels9110917)
Supplement: Supplementary file 1 [file gels-09-00917-s001.zip › gels-2727949-supplementary.pdf]

Highlights

- Residual mercaptopropionic acid strongly influences thiol-ene gelation time
- Premixing thiol and acrylate allows high polymer weight fraction hydrogels
- Freezing thiol and acrylate mixtures allows storage for greater than one month without gelation

Supporting Information

Factors That Influence Successful Base Catalyzed Thiol-ene Hydrogel Synthesis

Nolan J. Morrison, and Brandon M. Vogel*

Storage and Loss Moduli vs. Time Plot

The following plot displayed in **Figure S1** was obtained via dynamic rheology. The sample that produced the raw data was a 35 wt% hydrogel synthesized with a buffer pH of 7.4. The gelation time was determined to be the second intersection of the moduli, where the storage modulus rapidly increases.

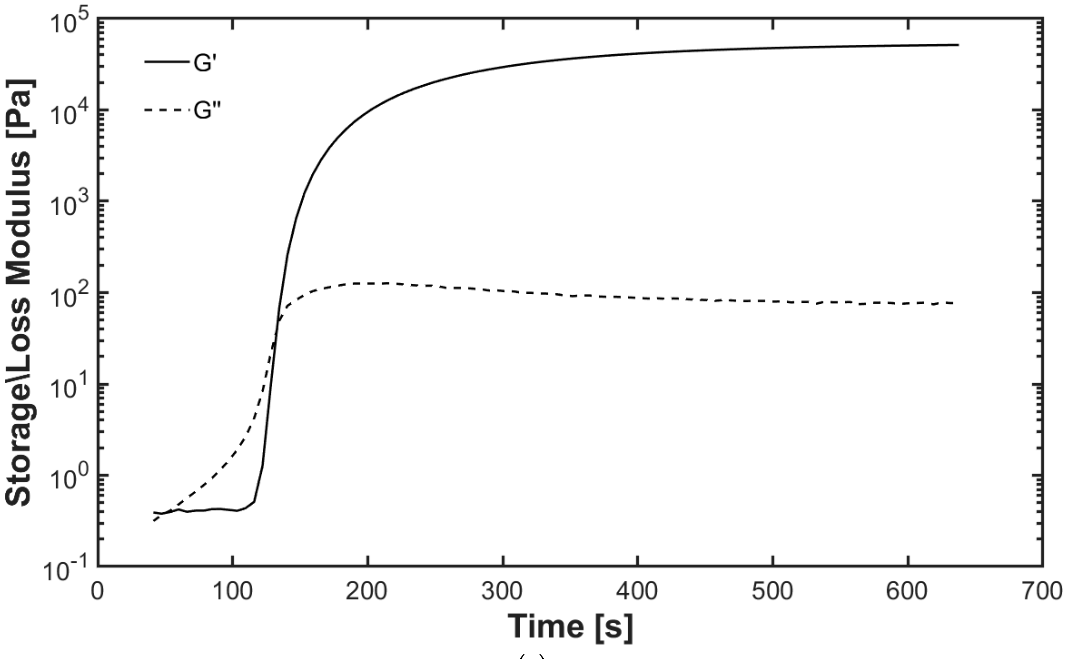

(a)

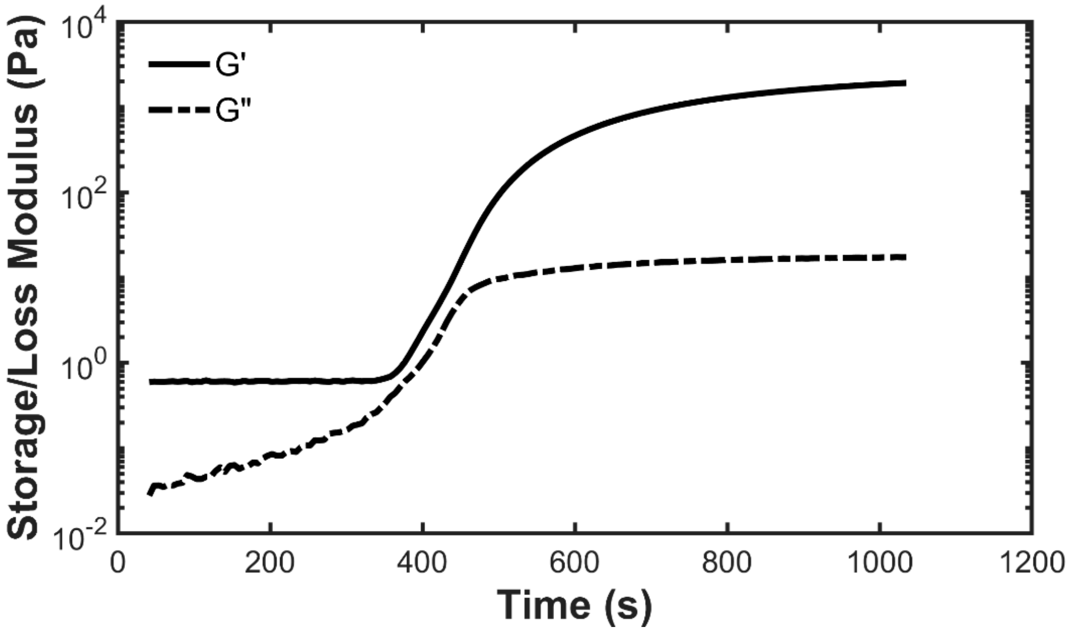

G'
G"
Storage/Loss Modulus (Pa)
Time (s)

(b)

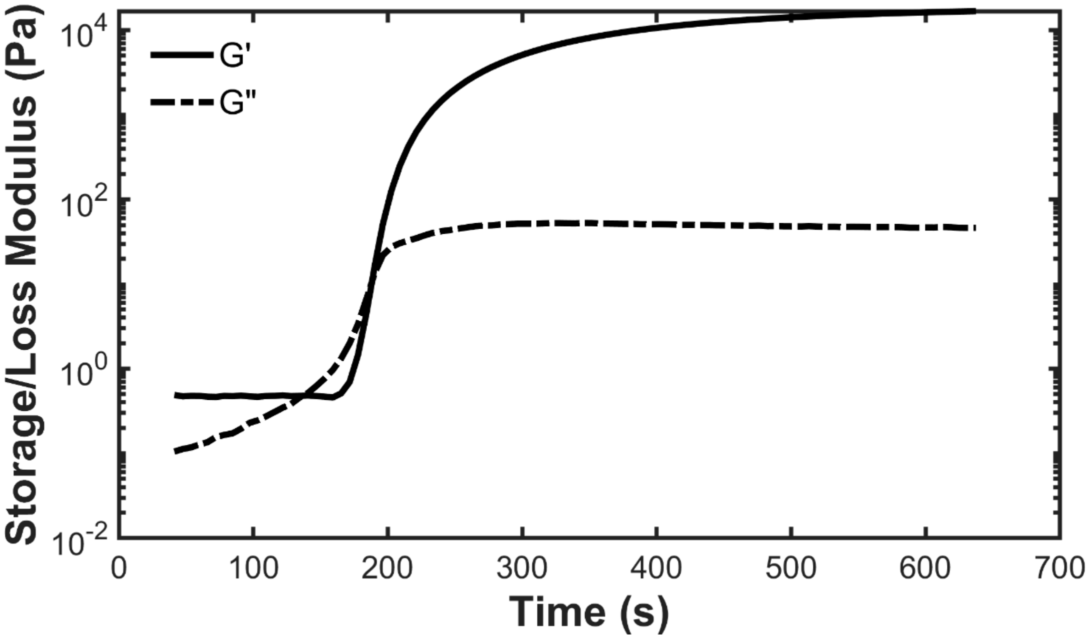

G'
G"
Storage/Loss Modulus (Pa)
Time (s)

(c)

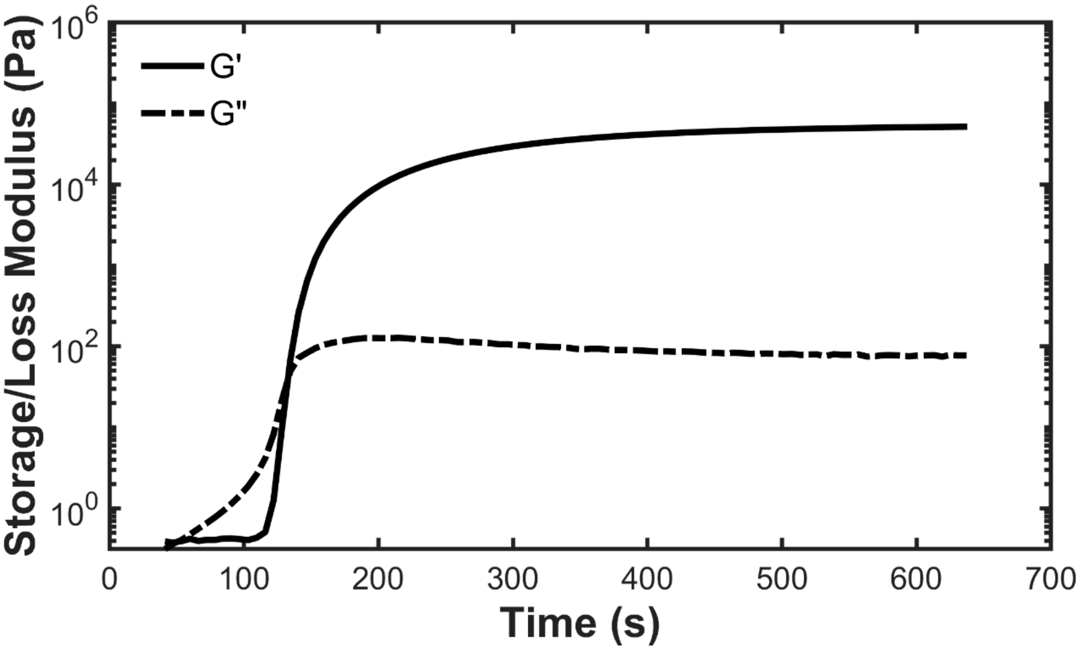

Storage/Loss Modulus (Pa)
Time (s)
G'
G''
10^6
10^4
10^2
10^0
0
100
200
300
400
500
600
700

(**d**)

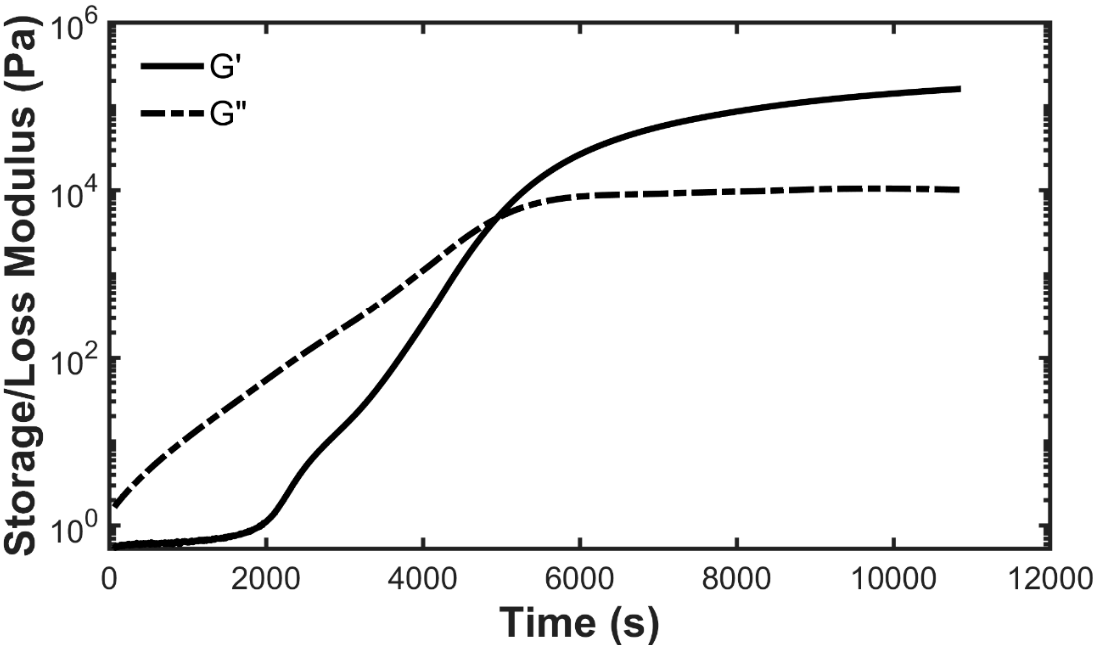

Storage/Loss Modulus (Pa)
Time (s)
G'
G''
10^6
10^4
10^2
10^0
0
2000
4000
6000
8000
10000
12000

(**e**)

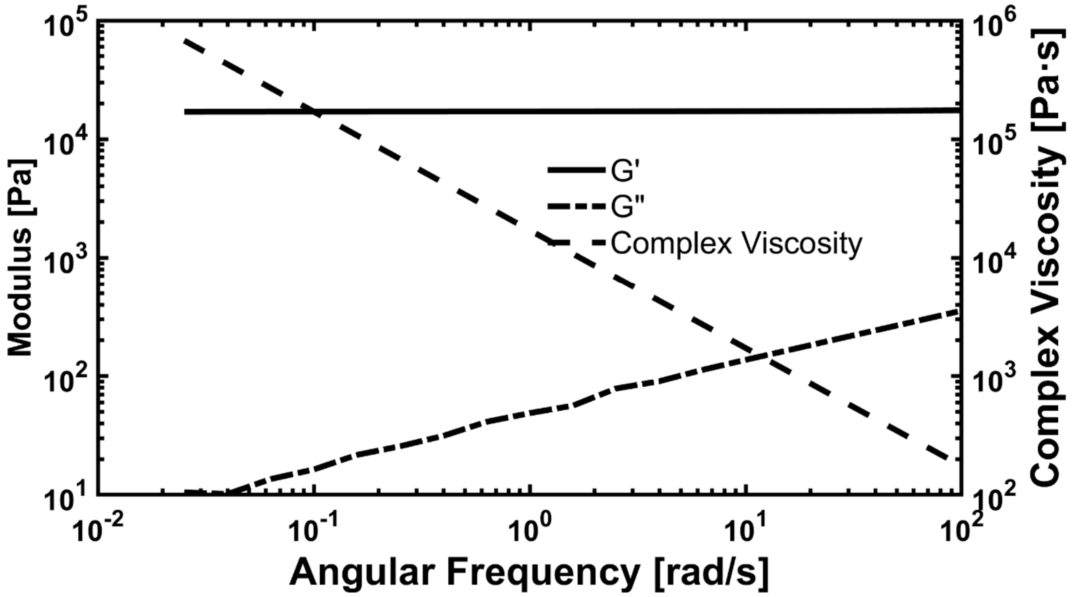

(f)

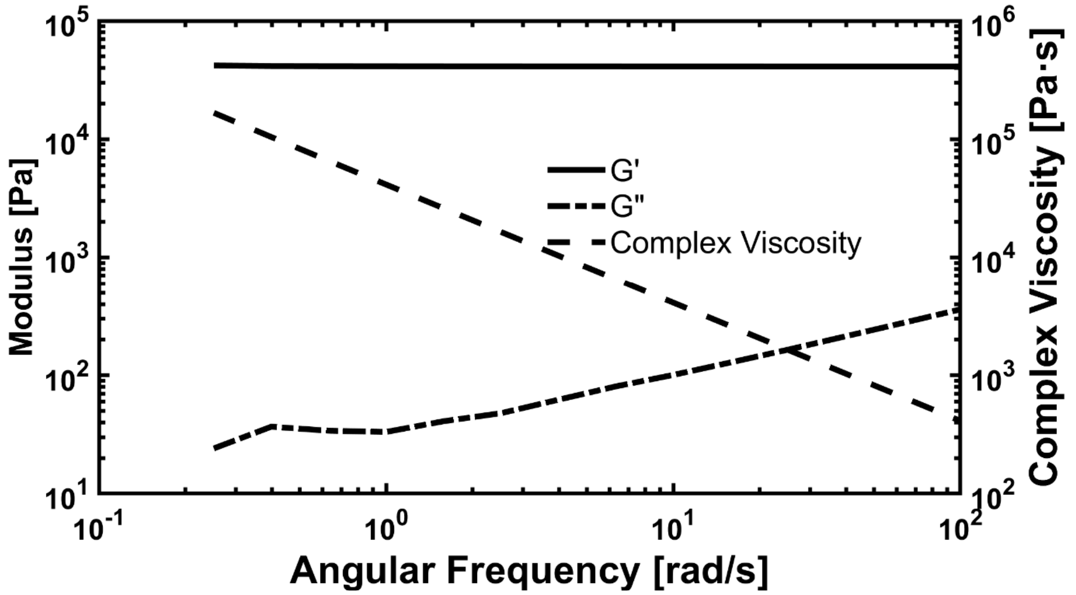

(g)

Figure S1. (**a**) Storage modulus (G') and loss modulus (G") vs. time for a 35 wt% hydrogel synthesized with a buffer pH of 7.4. (**b**) Storage modulus (G') and loss modulus (G") vs. time for a 15 wt% hydrogel synthesized with a buffer pH of 7.4. (**c**) Storage modulus (G') and loss modulus (G") vs. time for a 25 wt% hydrogel synthesized with a buffer pH of 7.4. (**d**) Storage modulus (G') and loss modulus (G") vs. time for a 35 wt% hydrogel synthesized with a buffer pH of 7.4. (**e**) Storage modulus (G') and loss modulus (G") vs. time for a 90 wt% hydrogel synthesized with a buffer pH of 7.4. (**f**) Storage modulus (G') and loss modulus (G") vs. angular frequency for a 25 wt% hydrogel synthesized with a buffer pH of 7.4. (**g**) Storage modulus (G') and loss modulus (G") vs. angular frequency for a 35 wt% hydrogel synthesized with a buffer pH of 7.4.

Full ETTMP Titration

A 0.09 M solution of ETTMP in DI H_2O was prepared in a beaker, and a 0.1 M NaOH solution was titrated in 0.1 ml increments until the first equivalence point was observed. Following the first equivalence, the NaOH was added in 1 ml increments until the total volume reached 200 ml, ensuring the second equivalence was reached. The pH of the solution was analyzed using a digital pH probe and recorded after each addition of NaOH. The full titration was completed for impure ETTMP (PCH = 0 cm) and purified ETTMP (PCH = 6.35 cm) and can be seen in **Figure S2.**

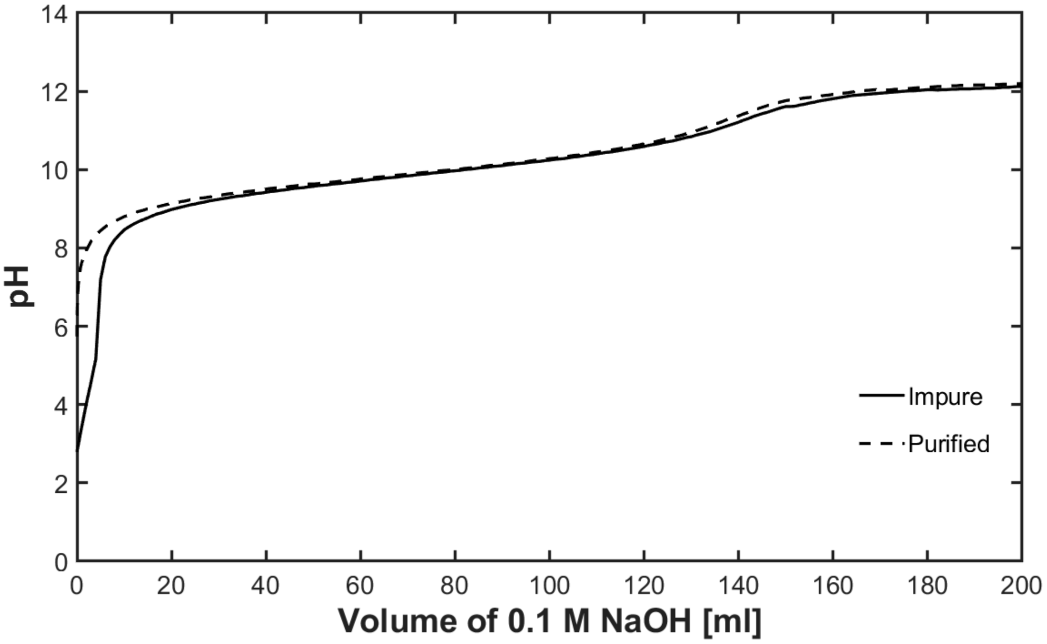

Figure S2 Full weak acid-strong base titration of impure ETTMP and ETMMP purified at a column height of 6.35 cm.

Mechanical Properties as a Function of Synthesis Parameters

The following sections provide data pertaining to the hydrogel mechanical properties, which include cross-link density, equilibrium modulus, and mesh size. We conducted this study to determine the effect of purification column height, buffer pH, and mixing type on the above properties. All samples were tested according to the procedure described in the "Instrumentation" section of the paper. Note that the properties are only a weak function of the specified parameter, and the large error bars are due to the sensitivity of the moduli data collected by the rheometer.

Purification Column Height

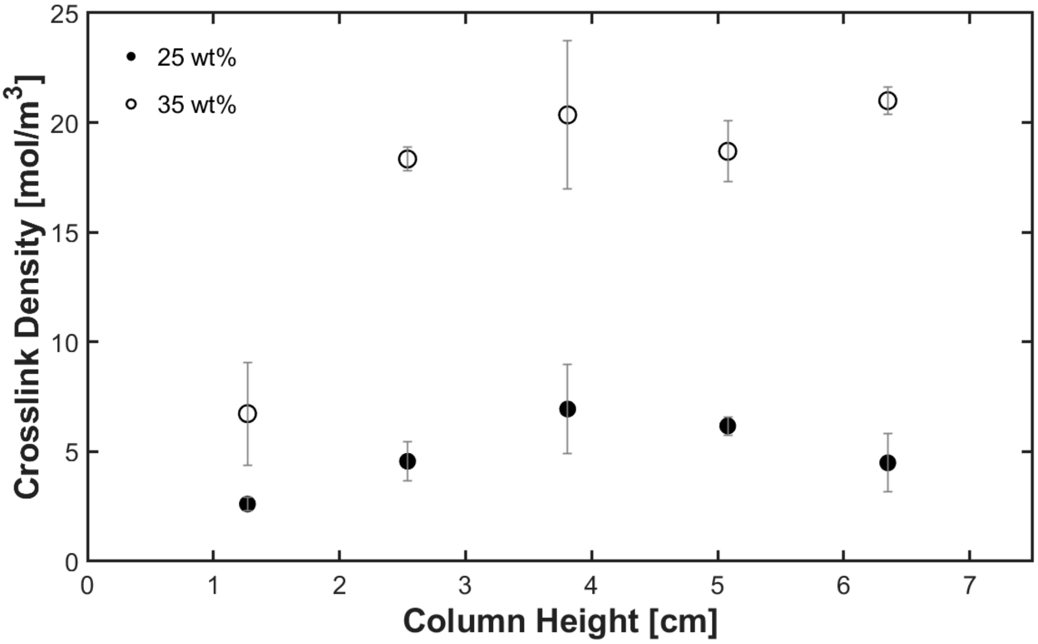

Figure S3 Cross-link density of 25 and 35 wt.% polymer concentration hydrogels as a function of purification column height, determined by dynamic rheology. Buffer pH and mixing type were kept constant at 7.4 and 15 s vortex, respectively. Error bars represent standard deviation, where n =3.

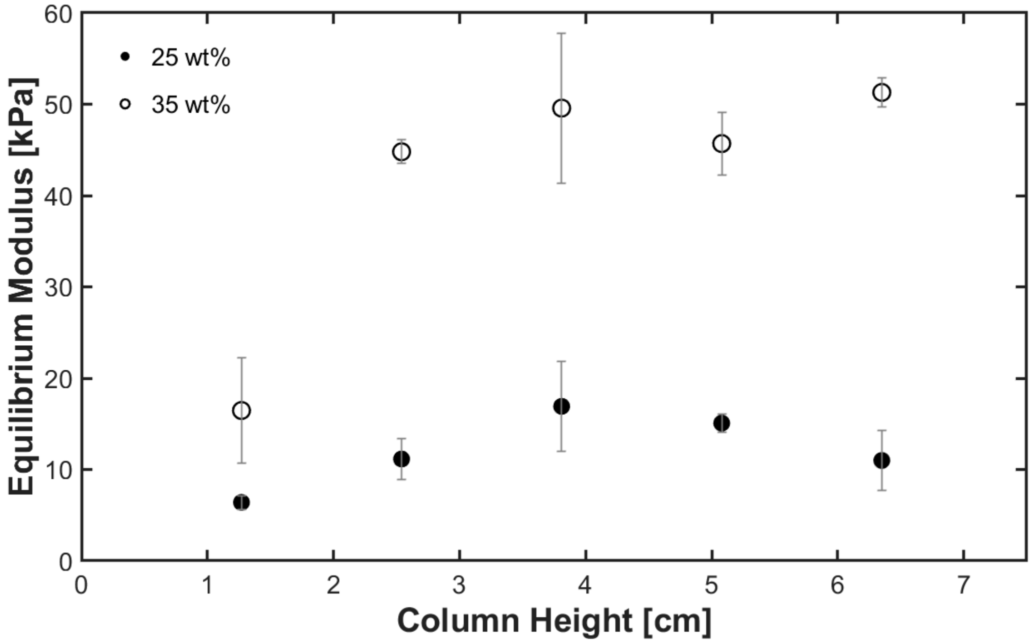

Figure S4 Equilibrium modulus of 25 and 35 wt.% polymer concentration hydrogels as a function of purification column height, determined by dynamic rheology. Buffer pH and mixing type were kept constant at 7.4 and 15 s vortex, respectively. Error bars represent standard deviation, where n =3.

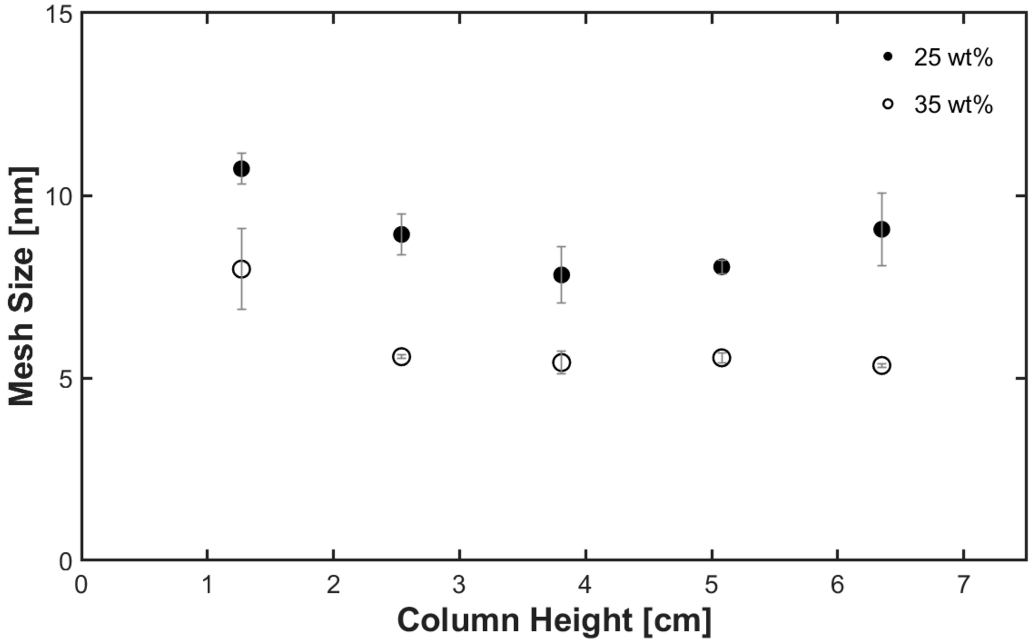

Figure S5 Mesh size of 25 and 35 wt.% polymer concentration hydrogels as a function of purification column height, determined by calculation from dynamic rheology data. Buffer pH and mixing type were kept constant at 7.4 and 15 s vortex, respectively. Error bars represent standard deviation, where n =3.

Buffer pH

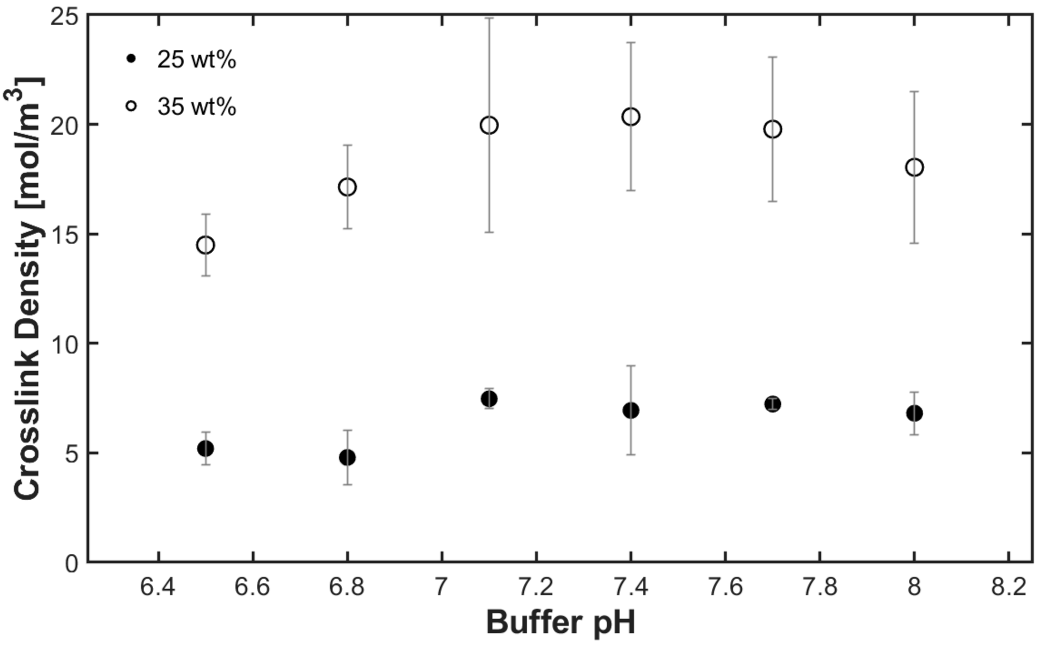

Figure S6 Cross-link density of 25 and 35 wt.% polymer concentration hydrogels as a function of buffer pH, calculated from dynamic rheology data. Purification column height and mixing type were kept constant at 3.81 cm and 15 s vortex, respectively. Error bars represent standard deviation, where n =3.

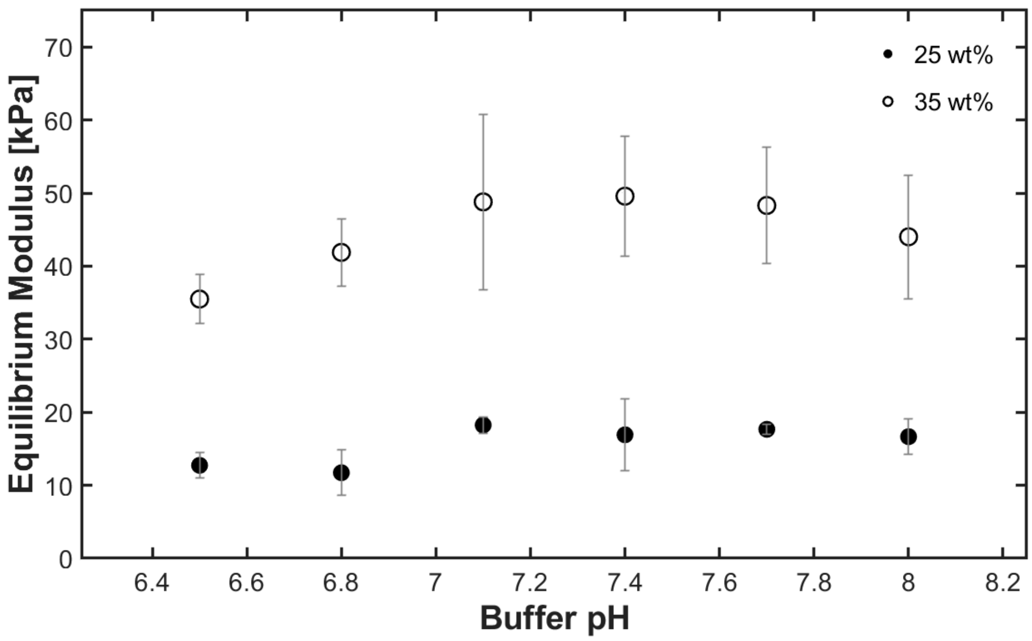

Figure S7 Equilibrium modulus of 25 and 35 wt.% polymer concentration hydrogels as a function of buffer pH, measure by dynamic rheology. Purification column height and mixing type were kept constant at 3.81 cm and 15 s vortex, respectively. Error bars represent standard deviation, where n =3.

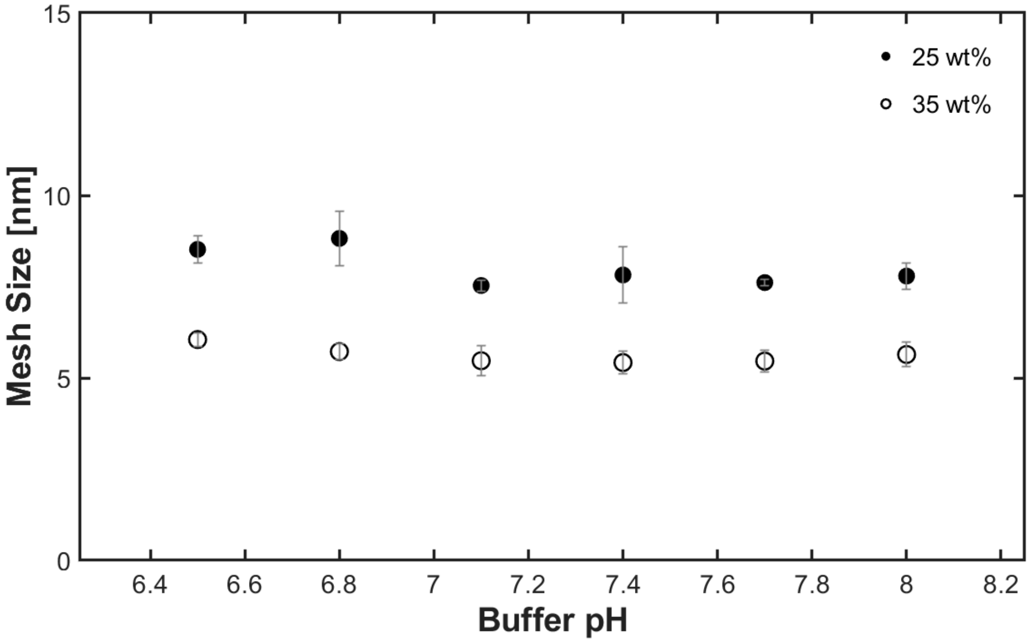

Figure S8 Mesh size of 25 and 35 wt.% polymer concentration hydrogels as a function of buffer pH, calculated from dynamic rheology data. Purification column height and mixing type were kept constant at 3.81 cm and 15 s vortex, respectively. Error bars represent standard deviation, where n =3.

Mixing Type

Table S1. Mechanical properties of 25 wt.% polymer concentration hydrogels as a function of mixing type, determined by dynamic rheology. Purification column height and buffer pH were kept constant at 3.81 cm and 7.4, respectively. Error represents standard deviation, where n =3.

Mixing Time [s]	Mixing Type	Cross-link Density [mol/m^3]	Mesh Size [nm]	Equilibrium Modulus [kPa]
5	Hand	5.29 ± 0.39	8.46 ± 0.20	12.93 ± 0.95
10	Hand	5.27 ± 1.00	8.52 ± 0.59	12.85 ± 2.44
15	Vortex	6.94 ± 2.03	7.82 ± 0.78	16.91 ± 4.94
25	Vortex	5.93 ± 2.06	8.27 ± 0.87	14.49 ± 5.04

Table S2. Mechanical properties of 35 wt.% polymer concentration hydrogels as a function of mixing type, determined by dynamic rheology. Purification column height and buffer pH were kept constant at 3.81 cm and 7.4, respectively. Error represents standard deviation, where n =3.

Mixing Time [s]	Mixing Type	Cross-link Density [mol/m^3]	Mesh Size [nm]	Equilibrium Modulus [kPa]
5	Hand	17.90 ± 3.22	5.65 ± 0.32	43.83 ± 7.91
10	Hand	15.97 ± 2.94	5.88 ± 0.38	39.10 ± 7.25
15	Vortex	20.35 ± 3.39	5.42 ± 0.31	49.57 ± 8.23
25	Vortex	19.01 ± 0.47	5.52 ± 0.05	46.59 ± 1.10

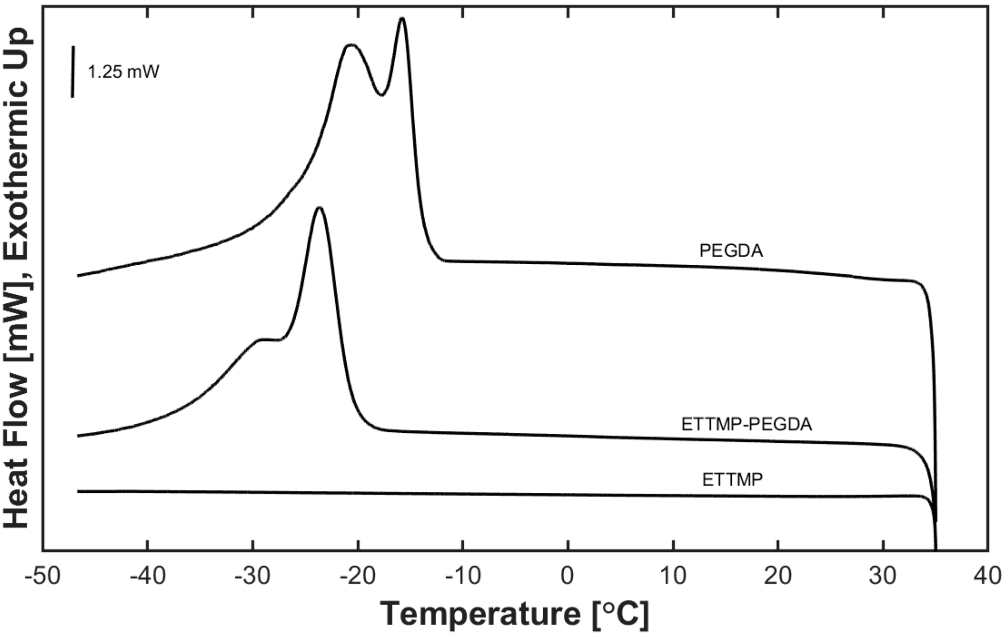

Figure S9 DSC thermograms of ETTMP, PEGDA, and a stoichiometric mixture of ETTMP and PEGDA. Samples were equilibrated at 35 ºC then cooled at a constant rate of 10 ºC/min.
